# Chasing the Cup: A Comprehensive Review of Spinal Cord Injuries in Hockey

**DOI:** 10.7759/cureus.24314

**Published:** 2022-04-20

**Authors:** Mitchell Self, James H Mooney, John Amburgy, James T Houston, Mark N Hadley, Dean Sicking, Beverly C Walters

**Affiliations:** 1 Neurological Surgery, Thomas Jefferson University, Philadelphia, USA; 2 Neurological Surgery, University of Alabama at Birmingham, Birmingham, USA; 3 Neurology, University of Alabama at Birmingham, Birmingham, USA; 4 Neurological Surgery, School of Mechanical Engineering, University of Alabama at Birmingham, Birmingham, USA

**Keywords:** comprehensive review, epidemiology, mechanisms, spinal cord injuries, hockey

## Abstract

Ice hockey is a high-speed sport with a high rate of associated injury, including spinal cord injury (SCI). The incidence of hockey-related SCI has increased significantly in more recent years. A comprehensive literature search was conducted with the PubMed, Medline, Google Scholar, and Web of Science databases using the phrases “hockey AND spinal cord injuries” to identify relevant studies pertaining to hockey-related SCIs, equipment use, anatomy, and biomechanics of SCI, injury recognition, and return-to-play guidelines.

Fifty-three abstracts and full texts were reviewed and included, ranging from 1983 to 2021. The proportion of catastrophic SCIs is high when compared to other sports. SCIs in hockey occur most commonly from a collision with the boards due to intentional contact resulting in axial compression, as well as flexion-related teardrop fractures that lead to spinal canal compromise and neurologic injury. Public awareness programs, improvements in equipment, and rule changes can all serve to minimize the risk of SCI.

Hockey has a relatively high rate of associated SCIs occurring most commonly due to flexion-distraction injuries from intentional contact. Further investigation into equipment and hockey arena characteristics as well as future research into injury recognition and removal from and return to play is necessary.

## Introduction and background

Approximately 10,000 Americans suffer from a catastrophic cervical spinal cord injury every year [1]. Up to 8% of all spinal cord injuries since 2005 have been sports-related [2]. Hockey is a high-speed sport with associated aggressive play and hard hits. Due to these characteristics, hockey players are prone to a high incidence and wide variety of injuries. Prior to 1980, ice hockey was a rare cause of spinal cord injury (SCI), however, the incidence has increased dramatically in more recent years [3]. The aim of the current comprehensive review is to summarize the epidemiology and injury mechanisms involved in spinal cord injury resulting from ice hockey and to explore how to potentially reduce these injuries.

## Review

Materials and methods

A literature search was performed using PubMed, Medline, Google Scholar, and Web of Science (July 12, 2021) with the following search phrase: “hockey AND spinal cord injuries”. Titles and abstracts were screened, relevant full-text articles were reviewed by independent authors, and the bibliographies of the full-texts were surveyed for additional pertinent studies. The primary areas of interest were spinal cord injuries (SCIs) in hockey players, the equipment used by hockey players, epidemiology and mechanisms of hockey-related SCI, anatomy and biomechanics of SCIs in hockey, injury recognition, and return-to-play guidelines. Studies of traumatic brain injuries, non-SCIs, and sports other than hockey were excluded.

Results

**Figure 1:**
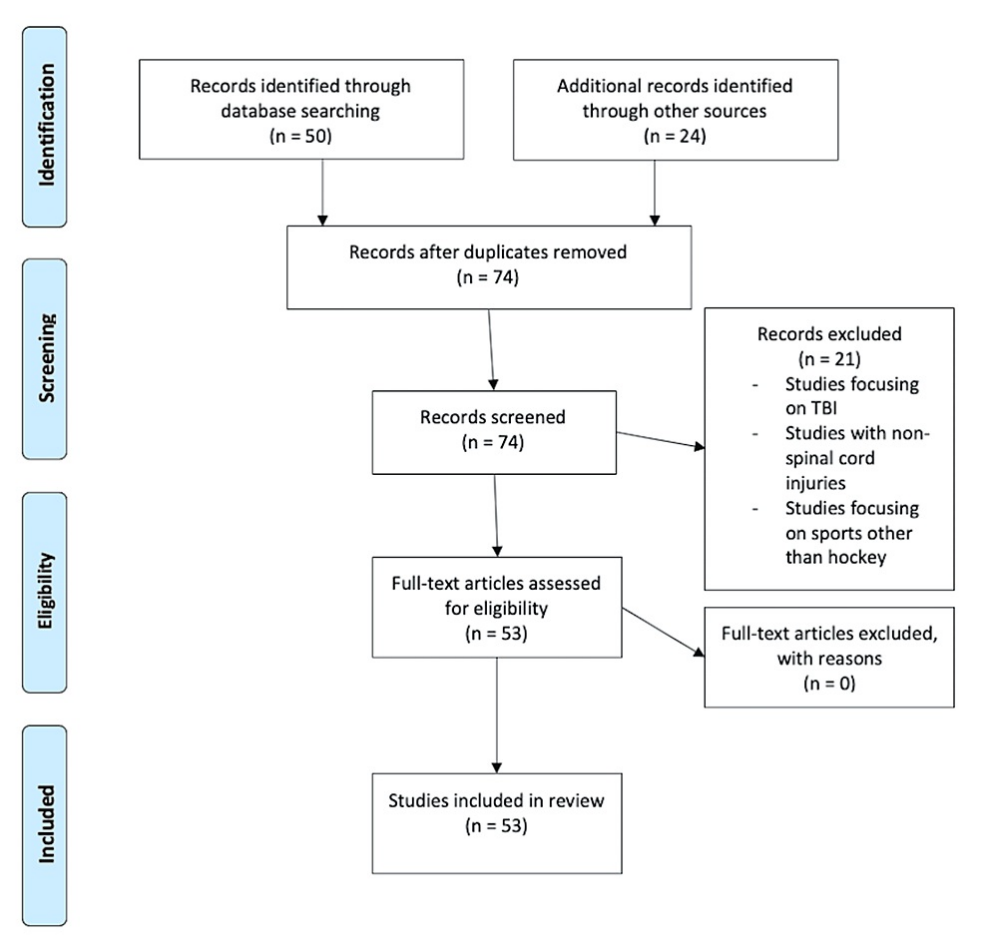
Flowsheet demonstrating the review process

The literature search yielded a total of 50 studies ranging from 1983 to 2021, from which 21 were excluded based on a review of abstracts. Studies focusing on traumatic brain injuries, non-SCIs, and sports other than hockey were all excluded. After a review of the article and their reference lists, an additional 24 studies were included based on relevance and ability to enhance knowledge on the topic. Ultimately, 53 studies underwent a comprehensive review for inclusion (Figure 1).

Hockey SCIs: Incidence/Prevalence

Sport-related injuries are the second most common cause of SCI in the first 30 years of life with approximately 7% of all new cases of SCI resulting from athletic activities [4]. Ice hockey injuries result in more than 18,000 emergency department visits each year [5]. Gerberich et al. found an overall injury rate of 75 injuries per 100 players [6]. While the number of catastrophic injuries in hockey is comparatively low, the incidence per 100,000 participants is high [4]. When compared with American football, the rate of paralysis due to SCI is at least three times higher in Canadian hockey [7]. Injuries most commonly occur in the high school to the college-aged group, i.e., players 16-20 years of age [8]. In fact, the rate of “catastrophic” injury caused by hockey in American high school-aged players is significantly higher than that caused by football in American high school players (2.56 injuries per 100,000 vs 0.68 injuries per 100,000) [9]. Injuries are much more common in games than in practices due to increased aggressiveness and speed [10-11].

Wennberg et al. showed that around 75% of the players suffering an SCI during hockey games suffered some form of a neurological deficit with close to one-third of these players being wheelchair-bound for life [10]. Other studies have shown that the majority of cervical fractures secondary to ice hockey are associated with permanent paralysis below the level of the injury [12-13].

Mechanisms of Injury

Severe SCIs in sports occur most commonly through head-first impacts resulting in axial compression [14]. Boden et al. found that 9% of all hockey injuries occur in the spine and similar studies show that over 60% of hockey-related spinal injuries result from a collision with the boards [15-16]. Additionally, close to 35% of injuries result from players being pushed or checked from behind [17].

For the average player, a cervical spine injury is likely if the peak compressive force on the upper neck is greater than 4000 N or if a force of 1100N has a pulse duration longer than 30 ms [18]. However, compression forces even lower, between 3611 and 8138 N, can lead to cervical quadriplegia in athletes [14]. A skater can reach 75% of the axial compressive load necessary for cervical spinal failure at only 1.8 m/s while skating speeds can easily exceed 12 m/s [16].

Injuries due to impact-loading occur within 2 to 19 milliseconds following head impact and prior to any significant head motion [19]. With the cervical spinal muscle reflex being between 50 and 54 ms [20], this timing of injury is two to three times faster than the cervical spinal muscle reflex [19]. Although muscles play a part in absorbing energy during impacts, with the neck able to absorb 0.6 kN more tensile loading with a tensed cervical spine [21], the protective effects of muscle may be diminished with compressive loading injuries [20]. In fact, some studies show that cervical muscle activation may cause higher peaks in compression and shear forces as well as longer durations of these forces [22].

Head rebound forces occur immediately after the initial impact and are observed in all cervical spine traumatic impacts [19]. In fact, a rebound can account for up to 963 ± 390 N in a padded surface impact [19]. Because of rebound, there are times when the forces on the cervical spine are greater than the forces measured on the head [19]. However, neck injuries due to axial compression during contact with a padded surface occur before the rebound of the head and torso [14].

Flexion and extension injuries occur at around 50% of the load required for an axial compression failure and the direction of force is only partially correlated with the injury [23]. This has been noted through analysis of pure flexion and combined flexion and compression injuries producing similar injury patterns [24]. These injuries can occur without any axial or lateral rotation and in the presence of low loading rates [24]. Of note, the impact angle is correlated with injury and may suggest that the orientation of the head, neck, and torso when compared to the impact surface may be of great importance [25]. Axial compression plus flexion leads to much more catastrophic injury to the spinal cord than axial compression with extension [26]. This increased risk of a catastrophic injury is due to the loss of protective cervical lordosis as well as the loss of alignment of the cervical vertebrae. In fact, joint loads are most affected by the pre-impact flexion angle of the neck [16,22].

Teardrop fractures occur when a flexed cervical spine undergoes high-energy axial compression such as occurs when being checked into the boards in hockey [27]. The mechanism behind these injuries is thought to be anterior vertebral column shear injury while the posterior vertebral column undergoes retropulsion ultimately resulting in spinal cord compression injury [27]. Multiple studies have shown that teardrop fractures occur most commonly at C5 and this is thought to be due to the steeper inclination of the uncinated process on this vertebra when compared to the adjacent vertebrae [27]. As with spinal cord concussions, spinal stenosis associated with increased age and individual variation in the space between the medial vertebral pedicle and compressed spinal cord may play a role in SCI mechanics [28-30].

Buckling is also common in compression injuries and occurs when a portion of the vertebral body compresses or bends but does not break all the way through. Buckling occurs after the axial compression load, causing non-physiologic motion between vertebrae with very little head rotation [14]. The impacts cause hyperflexion, most commonly at the C6-C7 and C7-T1 levels, and hyperextension above the C5 vertebra [31]. Increased vertebral mass and higher loading rates increase the likelihood of a higher-order mechanism of buckling, which leads to more complex deformations and a broader spectrum of cervical spinal injuries [32]. More complex deformations lead to greater increases in sagittal range of motion, lateral bending, as well as axial rotation in the cervical spine [31]. This is also demonstrated with higher energy impacts that cause more instability of the cervical spine in all directions [33].

Head pocketing occurs when the head impacts a padded surface, leading to decreased forces on the head but increased force on the cervical spine due to the neck being placed in axial compression for longer periods of time [34]. Although head pocketing may increase the risk of cervical SCI, it is not necessary for the injury to occur during the impact [19]. Some injuries result from a constrained cervical spine in the absence of a pocketing surface [19]. Additionally, impacts with padded surfaces produce significantly larger force impulses to the neck resulting in a much greater frequency of cervical spine injuries [25]. During these impactful events, the neck is a recipient of a significantly greater proportion of the torso’s momentum [25]. This leads to a greater frequency of injury in padded surface impacts when compared to rigid surface impacts [25]. Consequently, cervical spinal injuries in hockey are often attributed to the rapid deceleration of the head with the continued forward movement of the torso [35]. The padded impacts also caused a delay in the time of injury occurrence when compared with the rigid surfaces [20].

Potential for Injury Reduction

Despite the recognition that sports are a significant contributor to SCIs, methods to reduce and prevent injuries based on scientific evidence are lacking in ice hockey [36-37]. The most commonly reported cause of injury is intentional contact, followed by unintentional contact and equipment-related injury [5]. One explanation for this may be the increased allowances in playing style afforded to a player wearing a helmet [9]. Rigorous enforcement of rules may thus mitigate injuries resulting from increased aggression [10].

Some believe increased public awareness of the problem with programs directed at parents of hockey participants will help reduce injury numbers [38]. In fact, the slight drop in the annual incidence of hockey spinal injuries has been attributed to intense programs, such as the “Heads Up, Don’t Duck” campaign [5], implemented to focus on injury prevention [3]. These programs also propose that physicians encourage the use of proper helmets as well as provide supervision at hockey games with an understanding of acute care of SCIs [38]. Other programs push for prevention through proper strength and flexibility training [39]. One review article examined the efficacy of cervical spine muscle strength training for the prevention of hockey cervical SCIs finding a possible reduction in velocity and acceleration of the head during body impact with strength training. However, cervical muscle strengthening was not found to have a correlation with damage caused to the cervical spine after injury [40].

Creating equipment that reduces injury incidence first requires an understanding of not only the mechanism of injury and the tissue response to the injury but also the requirements of the players [41]. Hockey is a sport with a high demand for lightweight protective materials needed for high-contact scenarios [41]. When combined with speeds of up to 30 mph on a near-frictionless surface, this creates a challenging and unique protective necessity [42]. Because the pre-impact flexion angle of the neck is highly correlated with the joint loads on the neck, equipment to reduce or eliminate flexion angles should be investigated [22]. It may be possible to create a wearable strap or padding system that would reduce neck movements in players once a certain threshold is reached, similar to that created for football players [43].

Over the last 50 years, helmets and face masks are among the greatest changes to the sport of hockey [9]. Although head protection may decrease the incidence of traumatic brain injuries, it may have the unintended consequence of increasing the risk of catastrophic spine injuries [4]. While helmet padding has been found to decelerate the head-on impact, this places the burden on the cervical region to decelerate the torso, increasing the risk for SCI [41]. Reducing the number of concussions should not have to be traded for an increased rate of catastrophic SCI. In addition to the increased forces on the neck generated by helmet padding, the increased risk of a catastrophic injury may also partially result from the increased sense of invincibility that protective gear engenders in players [25,41]. Increased aggression can be associated with 80-90% of hockey injuries occurring in games as opposed to practice [11]. Smaller rinks may also lead to an increased likelihood of collisions with the boards resulting in higher injury rates [44]. Because of the increased rate of contact-related injuries when body checking is allowed, rules further limiting aggressive checking should be further implemented [5].

Also unique to the sport of hockey are rigid boards and hard goalposts [42]. Characteristics of the arena such as having more flexible boards and glass have been shown to reduce the risk of injury by almost one-third [45]. It is unknown if this increased risk with rigid boards is due to friction between the boards or not [44]. Because the lower limit of axial compression force resulting in quadriplegia in athletes is about 3600 N, investigations into more flexible boards that can reduce loads below this threshold are needed [14]. Poutiainen et al. showed that more flexible boards resulted in lower peak force and allowed for greater stopping distance [46]. Stopping distance is inversely correlated with resulting force and therefore a smaller injury risk [46]. Poutiainen et al. also showed more flexibility in a single framed system when compared with dual framed systems [46]. The mechanism of decreased injury with more flexible boards and framing systems should be investigated and should be further correlated with helmet studies and pocketing injuries to reduce the catastrophic injury incidence in ice hockey. It may be possible to create a novel hockey wall system that moves with impact, accepting some of the compressive load and reducing the axial load on the cervical spine, as well as modifying equipment to diminish cervical flexion upon striking whatever surface surrounds the playing area.

Discussion

One effort to dramatically reduce injuries in sports is through reliable data collection and analysis [47]. In the ice hockey population, head, neck, and facial injuries are the most common reasons for presentation to the emergency department [48]. Cervical spinal injuries most commonly occur as flexion-distraction injuries. An athlete with a flexed neck may be checked from behind into the boards and the combination of flexion with distraction can lead to subluxation or translation resulting in a disruption in one or both facet joints [14,49]. This can reduce the diameter of the spinal canal causing direct compression of the spinal cord [50]. As the athlete moves parallel to the ground with an impact surface oriented perpendicular to the flexed neck, resultant axial compression is the mechanism responsible for the greatest risk of neck injury in hockey [14]. This mechanism is often seen in American football-related injuries where over half of the injuries resulting in permanent cervical quadriplegia can be attributed to axial compression [34].

Compression injuries lead to fractures most commonly in the cervical spine, an area that is not structurally supported by ribs or extensive large musculature, with more than half of all SCIs occurring in the cervical spine [14]. More specifically, most of these cervical SCIs occur between the C5 and C7 vertebrae, as this is the location of the lever arm between the lordosis of the cervical spine and the kyphosis of the thoracic spine [11]. Other fracture types, such as teardrop fractures and buckling, can result in complex deformations, cervical spinal instability, and SCI. While padded surfaces may diminish the risk of head injury and concussion, they can have the paradoxical effect of increasing axial compression forces to the cervical spine thus increasing SCI risk.

Improvements in public awareness programs for parents and players as well as the education of physicians and standardization of care for hockey-related cervical spine injuries will all help minimize the societal consequences of these injuries. Injury tracking with registries will additionally provide improved insight into specific injury mechanisms and associations leading to the development of more targeted interventions such as improved equipment, facilities, and game rules.

Lastly, injury recognition, removal from play, treatment, recovery, and return-to-play are of paramount importance. In regard to return-to-play in the case of a cervical spine injury, the stakes are particularly high. The risk of a catastrophic spinal injury after returning to ice hockey needs to be addressed thoroughly on a case-by-case basis and prior guidelines have been developed to stratify patients into categories of risk based on their clinical presentation [51-52]. Despite agreement on fundamental criteria such as complete pain freedom, full cervical range of motion, full strength, and no residual neurologic deficits, there is still no consensus on the optimal management of cervical spine injuries in most sports, let alone ice hockey specifically [53]. Future research should be directed at improved injury recognition technologies as well as the development of more cohesive and standardized return-to-play guidelines.

## Conclusions

Hockey is a sport with a high incidence of catastrophic injuries related to its high speed and aggressive nature. SCIs are the most devastating of the injuries that occur in hockey and most of these are due to a player with a flexed neck being checked into the boards from behind. Axial compression is the main mechanism of injury that leads to multiple fractures and instability patterns in the cervical spine. Methods to reduce the incidence of SCIs in hockey have not been extensively studied. Helmets seem to be correlated with a decreased incidence of head injury but an increased incidence of severe SCIs. Helmet characteristics that may maintain the reduction in head injuries in addition to reducing cervical SCIs should be further investigated. Reducing the flexion angle of the neck could also reduce the incidence of hockey SCI and methods to alleviate neck flexion should be investigated further. Larger arenas and arenas with flexible boards reduce the risk of injury and these injury reduction possibilities should be further investigated through biomechanical testing of different board characteristics.

Further research into injury recognition, removal from play, and the development of more validated return-to-play criteria based on individualized risk-stratification will all lead to improved management of patients with hockey-related cervical spine injuries and the prevention of future injuries.
